# Evaluation of Chimerism Testing by Next-Generation Sequencing Using Insertion/Deletion Markers: Analytical Validation and Examples of Clinical Utilization

**DOI:** 10.1016/j.jmoldx.2026.04.002

**Published:** 2026-04-22

**Authors:** Shannon Dutterer, Christle Moore, Maya Giddens, Juliet Smith, Jenifer Williams, Jessica Magwood, Jeane Silva, Cathi Murphey, Valia Bravo-Egana

**Affiliations:** ∗ Wellstar MCG Health, Augusta, Georgia; † Baylor University Medical Center, Dallas, Texas; ‡ Loma Linda University Medical Center, Loma Linda, California; § School of Public Health, Augusta University, Augusta, Georgia; ¶ Southwest Immunodiagnostics, Inc., San Antonio, Texas; || Department of Surgery, Medical College of Georgia, Augusta, Georgia

## Abstract

Post-transplant engraftment monitoring is essential to assess the risk of complications after allogeneic hematopoietic transplantation, such as graft failure and disease relapse. It is based on the analysis of the percentage of chimerism detected in the recipient. Chimerism analysis has been routinely performed using short tandem repeats (STRs). Next-generation sequencing (NGS) has made possible the use of other genetic markers, such as single-nucleotide polymorphisms and insertions/deletions (indels). This study evaluated the performance characteristics of the NGStrack assay from GenDx to verify its accuracy, sensitivity, and reproducibility. It was determined that the assay can detect DNA contributed by donor and recipient within the range of 0.5% to 99.5%. The results of this assay were compared with an STR-based method, and examples of clinical utilization of the assay were provided using patient cases, including chimerism analysis on different cell subsets. It was confirmed that chimerism analysis using indel markers provides an increased sensitivity with respect to the reported sensitivity of assays using STR. The sensitivity for STR-based methods is in the range of 1% to 5%, whereas the sensitivity for the indel NGS-based method assessed here is 0.5%. The increased sensitivity and precise correlation between chimerism results with clinical events validates the usefulness of this assay for early diagnosis of disease relapse and graft failure and allows for more precise clinical management.

Post-transplant (PTx) engraftment monitoring is an essential tool to assess the risk of complications after allogeneic hematopoietic transplantation, such as graft failure and disease relapse.^1^, ^2^, ^3^, ^4^ It is based on the analysis of the percentage of hematopoietic chimerism detected in the recipient and is usually expressed as the percentage of DNA derived from donor cells or the ratio of DNA derived from donor cells/the DNA derived from recipient cells.^5^

Patterns of engraftment may vary depending on the type of graft, the recipient's disease, the conditioning therapy, and the level of human leukocyte antigen (HLA) compatibility, among other factors.^2^^,^^6^ The success of hematological recovery after the transplant can be hindered by engraftment failure, infections, graft-*versus*-host disease (GVHD), incompatibilities, and low number of cells infused (particularly CD34^+^ and CD3^+^ cells). Serial analysis of chimerism allows for monitoring the engraftment success over time.^7^

The read-out of the test ranges from full chimerism, referring to a recipient with exclusively donor hematopoietic cells, to different percentages of mixed chimerism, which can be further categorized as transient, stable, or progressive, depending on the changes of recipient and donor hematopoietic cell ratios at different stages after the transplant.

Chimerism analysis has been routinely performed by determining alleles with different short tandem repeats (STRs) in several loci across the genome.^8^^,^^9^ However, recent advancements, such as next-generation sequencing (NGS), have made possible the use of other genetic markers, such as single-nucleotide polymorphisms and insertions/deletions (indels), that differ between recipient and donor, to determine if the DNA is donor or recipient derived.^4^^,^^10^

NGS comprises a variety of technologies that allow for parallel sequencing of millions of DNA fragments. The deep sequencing coverage obtained with NGS significantly improves the accuracy for detecting mutations and genomic variants.^11^^,^^12^ The high-throughput capabilities make these technologies cost-effective for detection of such mutations and variants.^13^ It also allows the interrogation of several genomic regions at the same time, making it ideal for chimerism testing, where a greater number of informative markers between the recipient and the donor increases the sensitivity of the analysis to detect engraftment failure and relapse.^14^^,^^15^

In this study, the performance of the NGStrack assay from GenDx (for research use only, San Diego, CA) was evaluated. The assay uses 34 indel biallelic markers covering 378 distinctive nucleotide positions across 18 chromosomes (Figure 1). The method relies on detection of inserted or deleted DNA fragments by sequencing the regions where these markers are located using NGS. The results are analyzed by the TRKengine software (GenDx). It uses pretransplant genotyping of recipient and donor to help with selection of informative markers as well as with quantification of chimerism percentages in PTx samples.

**Figure 1:**
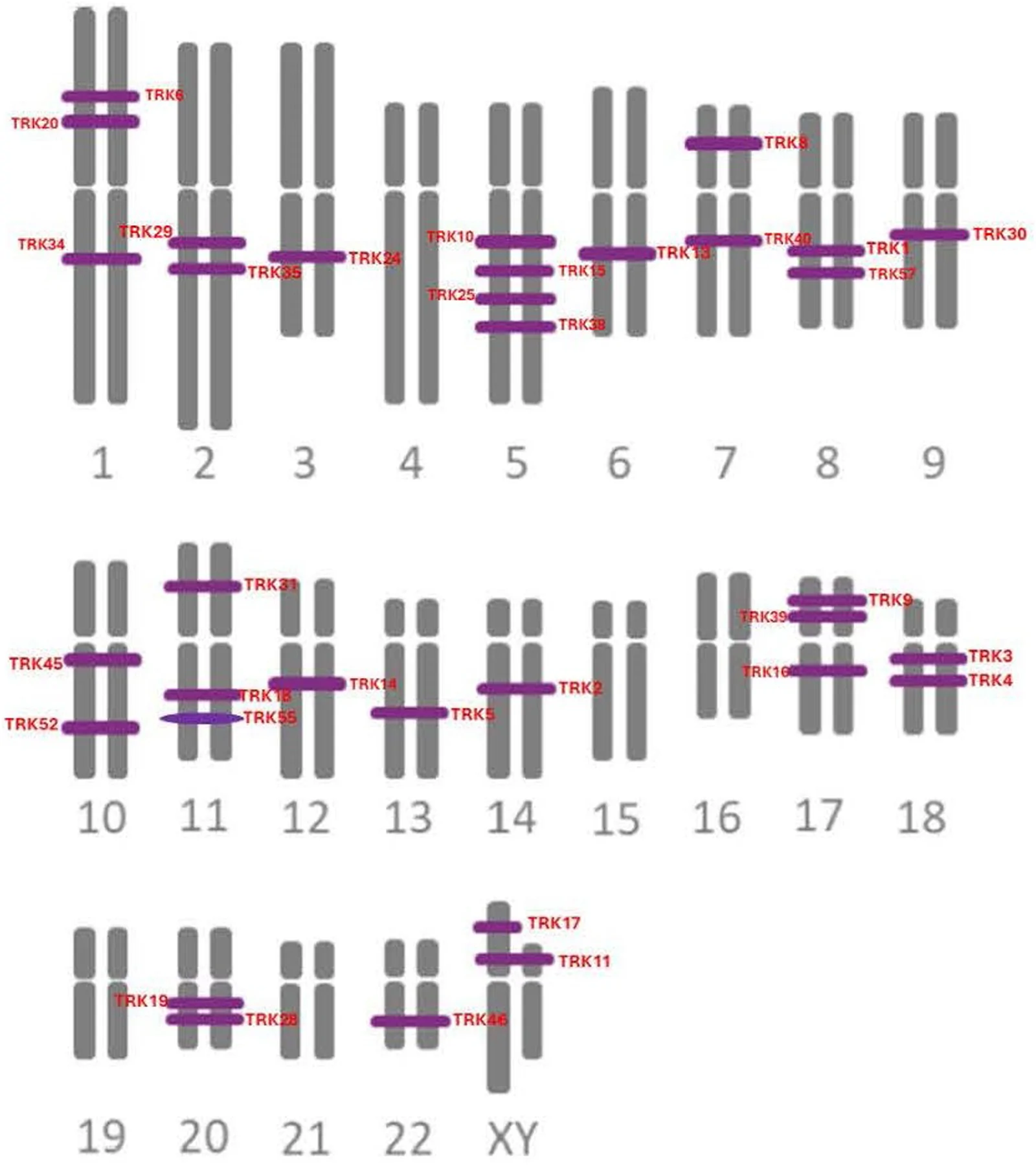
Distribution across chromosomes of the biallelic insertion/deletion markers interrogated by NGSTrack chimerism assay. Adapted from GenDx.

The performance characteristics of the assay were assessed to verify its accuracy, sensitivity, and reproducibility. Also, the results of this assay were compared with those of other assays, including an STR-based method. Last, examples of clinical utilization of this assay are provided using patient cases, including chimerism analysis on different lineage-specific cell subsets. Analysis of chimerism in specific cell subsets increases sensitivity because smaller amounts of recipient cells can be detected in isolated cell subsets compared with unfractionated whole blood samples.^2^^,^^16^^,^^17^ Increased sensitivity allows for detection of relapse, GVHD, and other complications earlier than when the analysis is performed in unfractionated samples.

## Materials and Methods

### Samples and Experimental Design

**Figure 2:**
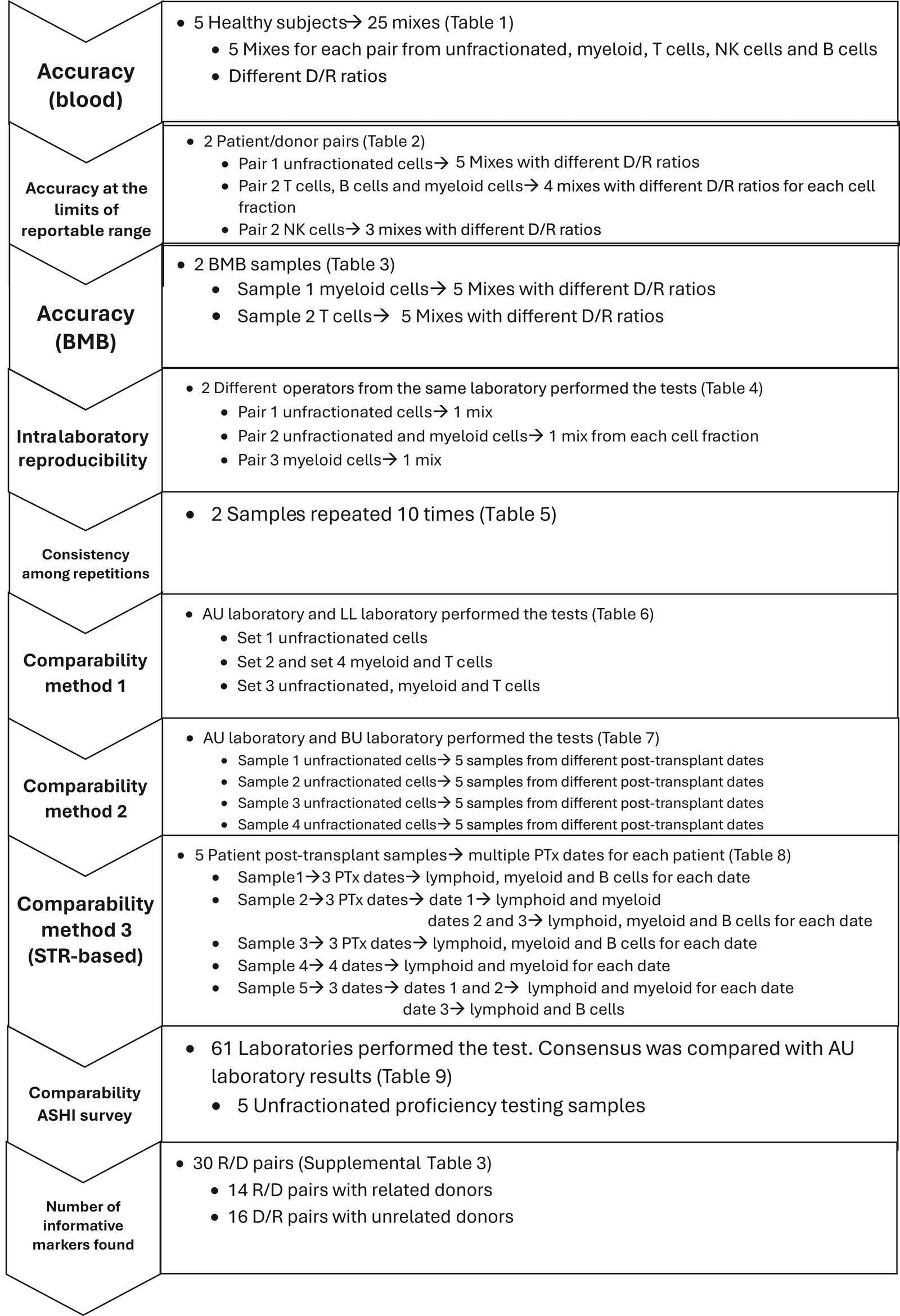
Study design flowchart. ASHI, American Society of Histocompatibility and Immunogenetics; AU, Augusta University; BMB, bone marrow biopsy; BU, Baylor University; D, donor; LL, Loma Linda; NK, natural killer; PTx, post-transplant; R, recipient.

A diagram detailing the experimental design followed for this validation is shown in Figure 2.

Different sets of samples were used to assess performance characteristics of the assay. First, DNA from five healthy individuals (three White, one African American, and one Middle Eastern) was mixed at different donor/recipient (D/R) ratios for a total of 25 mixes, including 5 mixes from unfractionated samples, 5 mixes from T cells, 5 mixes from B cells, 5 mixes from natural killer (NK) cells, and 5 mixes from myeloid cell fractions (Table 1).

**Table 1:** Chimerism Results of 25 Mixes Prepared from Unfractionated Cells and Four Lineage-Specific Cell Fractions (T Cells, B Cells, NK Cells, and Myeloid Fraction) from Five Healthy Individuals

Mixes	Cell type	D/R DNA mix ratio	Intended %	Chimerism result obtained, %	Absolute difference, %
Recipient 1 (Subject 1); donor 1 (Subject 2)	Unfractionated	75/25	75.0	74.9	0.12
	**Myeloid (CD33 + CD66b)**	75/25	75.0	74.8	0.23
	T cells (CD3)	75/25	75.0	75.5	0.46
	NK cells (CD56)	75/25	75.0	75.4	0.39
	**B cells (CD19)**	75/25	75.0	76.2	1.17
Recipient 2 (Subject 3); donor 2 (Subject 4)	Unfractionated	95/5	95.5	95.7	0.19
	Myeloid (CD33 + CD66b)	95/5	95.5	95.0	0.52
	T cells (CD3)	95/5	95.5	94.7	0.81
	**NK cells (CD56)**	95/5	95.5	96.7	1.22
	**B cells (CD19)**	95/5	95.5	95.8%	0.31
Recipient 3 (Subject 2); donor 3 (Subject 5)	Unfractionated	50/50	50.0	50.5	0.50
	**Myeloid (CD33 + CD66b)**	50/50	50.0	48.9	1.12
	T cells (CD3)	50/50	50.0	49.7	0.34
	**NK cells (CD56)**	50/50	50.0	47.6	**2.42**
	**B cells (CD19)**	50/50	50.0	56.5	**6.51**
Recipient 4 (Subject 4); donor 4 (Subject 3)	Unfractionated	30/70	30.0	30.8	0.78
	Myeloid (CD33 + CD66b)	25/75	25.0	25.7	0.70
	T cells (CD3)	25/75	25.0	26.2	1.15
	**NK cells (CD56)**	25/75	25.0	32.9	**7.87**
	**B cells (CD19)**	25/75	25.0	32.5	**7.47**
Recipient 5 (Subject 5); donor 5 (Subject 1)	Unfractionated	5/95	5.0	4.9	0.07
	Myeloid (CD33 + CD66b)	5/95	5.0	5.1	0.10
	T cells (CD3)	5/95	5.0	4.8	0.22
	**NK cells (CD56)**	5/95	5.0	5.8	**0.77**
	**B cells (CD19)**	5/95	5.0	14.3	**9.29**

Cell types in bold had below recommended DNA concentration, DNA quality, or both for the recipient, the donor, or both (see Supplemental Table S1). Absolute differences in boldface are >1.5%.

D, donor; NK, natural killer; R, recipient.

Two additional sets of mixes were used to assess accuracy and the limits of the reportable range in actual patients' and donors' samples. DNA from unfractionated cells from the first pair was used to prepare five mixes with different D/R ratios. Using DNA from the second pair, 4 mixes with different D/R ratios were prepared from the T cells, B cells, and myeloid fractions, and 3 mixes with different D/R ratios were prepared from NK cell fraction for a total of 20 mixes (Table 2).

**Table 2:** Chimerism Results of 20 Mixes Prepared from Unfractionated Cells and Four Lineage-Specific Cell Fractions (T Cells, B Cells, NK Cells, and Myeloid Fraction) from Two Donor-Recipient Pairs

Mixes	Cell type	D/R DNA mix ratio	Intended %	Chimerism result, %	Absolute difference, %
R/D pair 1	Unfractionated	96/4	96	95.98	0.02
		97/3	97	96.79	0.21
		98/2	98	98.05	0.05
		99/1	99	98.66	0.34
		99.05/0.5	99.50	99.55	0.05
R/D pair 2	T cell (CD3)	95/5	95.0	94.8	0.20
		98/2	98.0	98.1	0.10
		99/1	99.0	99.0	0.01
		99.5/0.5	99.5	99.6	0.10
	B cells (CD19)	95/5	95.0	94.2	0.80
		98/2	98.0	97.9	0.10
		99/1	99.0	98.6	0.40
		99.5/0.5	99.5	99.4	0.10
	Myeloid (CD33 + CD66b)	95/5	95.0	94.6	0.40
		98/2	98.0	97.9	0.10
		99/1	99.0	99.0	0.00
		99.5/0.5	99.5	99.4	0.10
	NK cells (CD56)	98/2	98.0	98.6	0.56
		99/1	99.0	99.1	0.13
		99.5/0.5	99.5	99.6	0.14

D, donor; NK, natural killer; R, recipient.

In another experiment, myeloid and T-cell fractions isolated from four bone marrow biopsy (BMB) samples were used to prepare 10 mixes (5 from each fraction) with different D/R ratios to assess the sensitivity of the test in this type of sample (Table 3).

**Table 3:** Chimerism Results of 10 Mixes Prepared from Myeloid Cells and T-Cell Fractions from Four BMB Samples

Mixes	Cell type	D/R mix ratio	Intended donor %	Chimerism result obtained, %	Absolute difference, %
Recipient 1 (BMB sample 1) donor 1 (BMB sample 2)	Myeloid cells	99.5/0.5	99.5	99.6	0.1
		95/5	95.0	97.2	2.2
		5/95	5.0	4.9	0.1
		50/50	50.0	47.2	2.8
		0.5/99.5	0.5	0.7	0.2
Recipient 2 (BMB sample 3) donor 2 (BMB sample 4)	T cells	1/99	1.0	0.8	0.2
		4/96	4.0	3.5	0.5
		50/50	50.0	49.3	0.7
		96/4	96.0	97.0	1.0
		99/1	99.0	99.2	0.2

BMB, bone marrow biopsy; D, donor; R, recipient.

To assess the reproducibility of the test, various approaches were considered. First, results obtained by two operators from the same laboratory were compared. For this experiment, four mixes from three pairs of healthy individuals (two mixes from unfractionated cells from pairs 1 and 2, and two mixes from myeloid cells from pairs 2 and 3) were prepared (Table 4). Second, the results of 10 repetitions from two donor-recipient pairs were compared (Table 5).

**Table 4:** Comparison of Results Obtained by Two Operators from the Same Laboratory

Pair	Mixes	Cell type	D/R ratio	Intended %	Chimerism result obtained by T1, %	Absolute difference between intended and T1 results, %	Chimerism result obtained by T2, %	Absolute difference between intended and T2 results, %	Absolute difference between T1 and T2 results, %
Pair 1	R (Subject 2)/D (Subject 5)	Unfractionated	50/50	50.0	50.5	0.5	50.7	0.7	0.2
Pair 2	R (Subject 4)/D (Subject 3)	Unfractionated	30/70	30.0	28.0	2.0	29.0	1.0	1.0
		Myeloid (CD33 + CD66b)	25/75	25.0	25.7	0.7	26.0	1.0	0.3
Pair 3	R (Subject 5)/D (Subject 1)	Myeloid (CD33 + CD66b)	5/95	5.0	5.1	0.1	5.0	0.0	0.1

D, donor; R, recipient; T1, technologist 1; T2, technologist 2.

**Table 5:** Chimerism Results of 10 Repetitions from Two Donor-Recipient Pairs

Chimerism results for sample 1, %	Absolute difference between intended (1%) and obtained results for sample 1, %	Chimerism results for sample 2, %	Absolute difference between intended (1%) and obtained results for sample 2, %
0.84	0.16	0.85	0.15
0.66	0.34	1.01	0.01
0.53	0.47	0.41	0.59
1.29	0.29	0.87	0.13
0.65	0.35	0.94	0.06
1.24	0.24	0.31	0.69
0.50	0.50	1.38	0.38
0.48	0.52	1.25	0.25
0.77	0.23	0.45	0.55
0.56	0.44	0.91	0.09

Statistics	Sample 1, %	Sample 2, %	
Average	0.75	0.84	
SD	0.3	0.3	
CV	39.1	40.0	

Comparability studies to assess precision and bias between methods were performed by comparing results obtained by other laboratories on patient samples. NGSTrack results for different cell subsets isolated from four patient samples were compared with results obtained by the Histocompatibility Laboratory at Loma Linda University Medical Center (Loma Linda, CA), using chimerism NGS assay from Devyser (Thermo Fisher Scientific, Waltham, MA) (Table 6). Similarly to the NGSTrack assay evaluated here, Devyser NGS assay uses indel markers, but although NGSTrack uses 34 markers distributed across 18 chromosomes, the Devyser NGS assay uses 24 markers across 17 chromosomes. Illumina (San Diego, CA) MiSeq platform is used for sequencing, and Devyser’s proprietary Advyser chimerism software version 3.1.2.0 is used for analysis.

**Table 6:** Comparison of Results Obtained by the LL Laboratory and the AU Laboratory

Sample	Sample type	Concentration, ng/μL	260/280 ratio	LL laboratory result, %	AU laboratory result, %	Absolute difference, %
Patient 1	Unfractionated	220.1	1.86	99.80	99.75	0.05
Patient 2	Myeloid	39.4	1.77	96.72	96.96	0.24
	T-cell CD3	67.2	1.83	82.11	82.52	0.41
Patient 3	Unfractionated	422	1.86	99.77	99.65	0.12
	Myeloid	185.5	1.86	99.94	99.94	0.00
	T-cell CD3	152.9	1.84	99.18	98.98	0.20
Patient 4	Myeloid	19.1	1.91	99.95	99.59	0.36
	T-cell CD3	80.3	1.9	34.40	34.99	0.59

AU, Augusta University; LL, Loma Linda.

Also, this study compared results for 20 patient samples provided by the Transplant and Genomics Core at Baylor University Medical Center (Dallas, TX) performed by this laboratory using the ScisGo chimerism MD kit (Scisco Genetics, Seattle, WA) (Table 7). The ScisGo assay is designed to identify both single-nucleotide polymorphism and indel markers using NGS on the Illumina MiSeq platform. The initial version comprised a panel of 37 single-nucleotide polymorphisms across 18 chromosomes and 12 indel markers across 10 chromosomes.^18^ Successive versions increased the number of markers, but the specific number is proprietary information. The version used to obtain the data presented here was version 5. The system uses ScisGo cloud software for analysis.

**Table 7:** Comparison of Results Obtained by the BU Laboratory and the AU Laboratory

Sample	Cell type	Concentration, ng/μL	260/280 ratio	AU results, %	BU results, %	Absolute difference, %
Sample 1						
Date 1	Unfractionated	69.5	1.81	14.32	14	0.32
Date 2	Unfractionated	112.5	1.86	48.38	49	0.62
Date 3	Unfractionated	106.8	1.86	33.63	35	1.37
Date 4	Unfractionated	93	1.87	99.89	100	0.11
Date 5	Unfractionated	86.2	1.84	89.69	90	0.31
Sample 2						
Date 1	Unfractionated	59.2	1.84	94.91	95	0.09
Date 2	Unfractionated	56.3	1.87	84.14	86	1.86
Date 3	Unfractionated	55.4	1.83	5.95	5	0.95
Date 4	Unfractionated	51.7	1.85	48.75	51	2.25
Date 5	Unfractionated	51.1	1.85	14.95	15	0.05
Sample 3						
Date 1	Unfractionated	35.1	1.88	9.53	10	0.47
Date 2	Unfractionated	38.6	1.76	3.11	3	0.11
Date 3	Unfractionated	105.6	1.82	90.33	90	0.33
Date 4	Unfractionated	60.5	1.85	76.44	75	1.44
Date 5	Unfractionated	49.5	1.87	61.31	60	1.31
Sample 4						
Date 1	Unfractionated	55.8	1.81	4.95	5	0.05
Date 2	Unfractionated	49.8	1.86	50.58	51	0.42
Date 3	Unfractionated	28.7	1.76	10.18	11	0.82
Date 4	Unfractionated	59.4	1.82	94.60	94	0.60
Date 5	Unfractionated	80.2	1.84	84.65	0.86	1.35

Five post-transplant dates for each patient sample were tested.

AU, Augusta University; BU, Baylor University.

In addition, the chimerism results of 40 samples performed using STR-based PCR method (PowerPlex 21; Promega, Madison, WI) done at the Southwest Immunodiagnostics Histocompatibility laboratory (San Antonio, TX) were compared with the results obtained in our laboratory using the NGSTrack assay (Table 8).

**Table 8:** Comparison of Results Obtained by the AU Laboratory Using NGSTrack and the SWID Using an STR-Based Method

Samples	Sample date	Lineage	Concentration, ng/μL	SWID results, %	AU results, %	Absolute difference, %
Sample 1	1	Lymphoid	10.5	26	28.10	2.10
		Myeloid	28.7	99	99.90	0.90
		B cell	11.8	98	98.10	0.10
	2	Lymphoid	20	51	43.20	7.80
		Myeloid	8	100	99.90	0.10
		B cell	29	98	98.50	0.50
	3	Lymphoid	26	35	32	3.00
		Myeloid	82	100	99.80	0.20
		B cell	68	98	98.80	0.80
Sample 2	1	Lymphoid	22.2	42	40.50	1.50
		Myeloid	16.8	100	99.40	0.60
	2	Lymphoid	103	97	96.90	0.10
		Myeloid	94	100	99.90	0.10
		Unfractionated	33	100	98.80	1.20
	3	Lymphoid	78	99	99.70	0.70
		Myeloid	56	100	99.90	0.10
		Unfractionated	45.4	100	99.90	0.10
Sample 3	1	Lymphoid	121	24	24.07	0.07
		Myeloid	133	100	99.84	0.16
		Unfractionated	60.5	78	75.80	2.20
	2	Lymphoid	12.7	19	18.08	0.92
		Myeloid	95	100	99.30	0.70
		Unfractionated	36	75	74.80	0.20
	3	Lymphoid	31	41	42.10	1.10
		Myeloid	84	100	99.60	0.40
		Unfractionated	19.7	91	91.75	0.75
Sample 4	1	Lymphoid	16.7	66	67.08	1.08
		Myeloid	19.5	100	99.70	0.30
	2	Lymphoid	16	67	67.20	0.20
		Myeloid	83	100	99.50	0.50
	3	Lymphoid	20.5	67	69.90	2.90
		Myeloid	86.9	100	99.70	0.30
	4	Lymphoid	53	90	89.60	0.40
		Myeloid	153	100	99.80	0.20
Sample 5	1	Lymphoid	9.9	69	68.70	0.30
		Myeloid	31.4	100	99.60	0.40
	2	Lymphoid	19	89	90.86	1.86
		Myeloid	147	100	99.32	0.68
	3	Lymphoid	73	100	99.80	0.20
		B cell	35	100	99.90	0.10

AU, Augusta University; STR, short tandem repeat; SWID, Southwest Immunodiagnostics Histocompatibility Laboratory.

Finally, this study tested five samples from a proficiency testing survey from the American Society of Histocompatibility and Immunogenetics and compared results with consensus obtained by 61 participants (Table 9).

**Table 9:** Comparison of Results Obtained by the AU Laboratory with Consensus Results from 61 Participants in a Proficiency Testing Survey from the American Society of Histocompatibility and Immunogenetics

Set	Sample type	Concentration, (ng/μL)	260/280 ratio	Survey result (median), %	AU result, %	Absolute difference, %
Recipient (EMO-61); donor (EMO-62)	Unfractionated 166	67.9	1.86	34.34	34.94	0.60
	Unfractionated 167	75.2	1.84	73.64	75.95	2.31
	Unfractionated 168	78.4	1.86	0.00	0.35	0.35
	Unfractionated 169	64.7	1.85	78.00	80.15	2.15
	Unfractionated 170	72.5	1.80	58.98	60.27	1.29

AU, Augusta University.

Thirty donor-recipient pairs (14 related and 16 unrelated) were examined to determine the average number of informative markers used by this assay; and to illustrate clinical utility, samples from three patients were tested over time.

The study was approved by the Institutional Review Board at Augusta University (Augusta, GA).

### DNA Extraction and Cell Subset Isolation

DNA was isolated from whole blood preserved in acid citrate dextrose for unfractionated analysis and from cell subsets previously isolated by positive selection using CD3, CD19, CD56, and a mix of CD33 and CD66b markers for T cells, B cells, NK cells, and myeloid fraction, respectively, according to the manufacturer's instructions (STEMCELL Technologies, Vancouver, BC, Canada). In brief, the method uses antibody complexes linked to EasySep magnetic particles (STEMCELL Technologies). The labeled cells remain on the sides of the tube attracted to an EasySep cell separation magnet, whereas the unwanted cells are poured off. Three washes of the cells are performed to increase purity. Cells are finally eluted in 350 μL of EasySep buffer. Purity of the different cell fractions is assessed by flow cytometry using CD2, different clones from those used for cell isolation of CD19 and CD56, and a mix of CD45, CD14, and a different clone of CD66b fluorescein isothiocyanate–conjugated antibodies for T cells, B cells, NK cells, and myeloid cells, respectively. On average, the purity for all cell lineages was >95%.

In all cases, DNA was isolated using Qiagen (Hilden, Germany) EZ1 QIAxpert system, which includes the EZ1 automated nucleic acid purification system and the QIAxpert spectrophotometer to measure DNA concentration and purity through the 260/280 ratio. Supplemental Table S1 shows DNA concentration and 260/280 ratios for these samples.

### NGSTrack Chimerism Assay

The manufacturer's protocol edition 3 from October 2022 was followed. Briefly, sample preparation starts by making dilutions of 20 ng/μL for all genotype samples and 10 ng/μL for all PTx monitoring samples. Then, 1.5 μL of each sample is added to a mixture of primers and enzyme to amplify the regions of interest. After amplification by PCR, the fragments are indexed and amplified by a second PCR. This is followed by pooling and cleaning steps, library quantification by Qubit fluorometer (Invitrogen, Eugene, OR), adjustment of concentration to reach 12 pmol/L that are loaded in the Illumina MiSeq, and sequenced using a MiSeq flow cell. The assay uses TRKengine software version 1.4.0.4070 for analysis using the FASTQ files generated by the sequencing data.

### PowerPlex 21 STR-Based Assay

The PowerPlex 21 assay allows for the co-amplification and fluorescent detection of 21 markers. The system utilizes PCR amplification of human STR regions, followed by capillary electrophoresis using Applied Biosystems 3150 Genetic Analyzer (Thermo Fisher Scientific), according to the manufacturer's protocol. Analysis was performed using ChimerMarker software version 3.1.8 (Softgenetics, State College, PA). The software helps to identify donor and recipient electropherogram peaks in PTx samples and calculates percentage chimerism and quality metrics.

### Statistical Analysis

Pearson correlation and linear regression analysis were utilized to assess the linear relationship between the intended chimerism and the chimerism results obtained. Absolute differences between the intended chimerism percentage and the chimerism percentage obtained were also calculated for each mix prepared.

Average, SD, and CV were calculated to assess the variability of 10 repetitions aimed to provide evidence of the reproducibility of the test.

Two-sample unequal variance *t*-test was used to compare the absolute differences between intended chimerism percentage and actual chimerism obtained between samples with and without optimal DNA concentration and/or quality. Paired *t*-test was used to compare results obtained using NGSTrack with results obtained with an STR-based method, as well as to compare the number of informative markers found between related and unrelated pairs.

## Results

### Assay Performance Characteristics

#### Linearity and Accuracy

**Figure 3:**
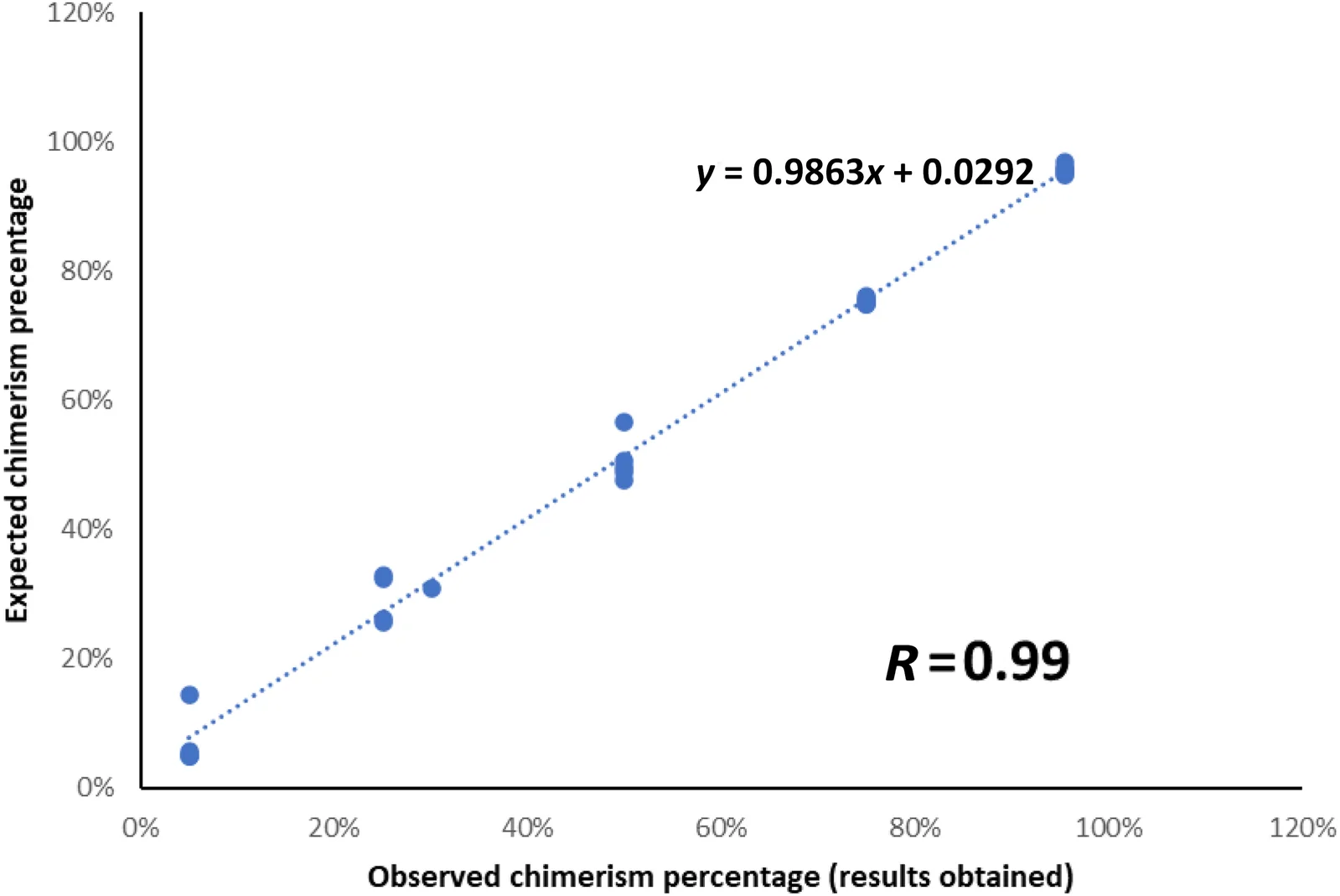
Correlation between the expected and observed chimerism results in 25 mixes prepared from unfractionated, myeloid cell, T cell, B cell, and natural killer cell fractions from five healthy individuals.

Chimerism analyses of 25 mixes prepared from five D/R pairs with different D/R ratios were performed from unfractionated cells and four lineage-specific cell fractions (T cells, B cells, NK cells, and myeloid fraction) (Table 1). A strong linear positive correlation was found between the expected and observed chimerism results, with a Pearson correlation coefficient (*R*) of 0.99 (*P* < 0.00001) (Figure 3).

Accuracy was determined by calculating the absolute difference between the intended percentages and the percentages of chimerism obtained. For pairs 1 and 2, all the differences were <1%, except for the B-cell fraction in pair 1 and the NK cell fraction in pair 2, with differences of 1.1% and 1.2%, respectively. However, for both tests, DNA concentration and 260/280 ratio were <10 ng/μL and <1.8, respectively, which are the values recommended by the manufacturer. DNA from these cell fractions was isolated from a relatively low number of cells, which compromised the yield. Similarly, for pairs 3, 4, and 5, all the differences were <1%, except for mixes where the DNA concentration and purity were below recommended. For pairs 3, 4, and 5, the differences in B cell and NK cell fractions were significantly impacted by the low DNA concentration and quality obtained.

In the tests performed from myeloid cells in pair 3 and T cells in pair 4, the absolute differences between the intended and the obtained chimerism percentages were slightly >1%, with 1.1% in both cases. The numbers of recipient cells in the myeloid fraction for pair 3 and in the T-cell fraction for pair 4 were both slightly lower because the cells from each individual had to be divided to prepare the different mixes.

Overall, in 16 of the 25 mixes (64% of the mixes), the differences were <1%, and in 20 of the 25 mixes (80% of the mixes), the differences were <1.5%. Only in five mixes, the absolute differences were >2%. Three of these mixes were B cells, and the other two were NK cells. In all of them, the DNA concentration, DNA quality, or both were below recommended.

To confirm the contribution of DNA concentration and quality to the test accuracy, the absolute differences between intended chimerism percentages and actual chimerism obtained were compared for samples with and without optimal DNA concentration and/or quality. This difference was statistically significant, with *P*= 0.018.

Notably, even in samples with suboptimal requirements, in some cases with exceptionally low concentration and purity, the absolute differences between the intended and the percentage chimerism obtained were acceptable, such as in the NK cell fractions of pairs 5 and 3, where they were 0.7% and 2.4%, respectively. Although this demonstrated the reliability of the system, it was evident that DNA concentration and quality within the recommended ranges were crucial for the accuracy of the assay.

**Figure 4:**
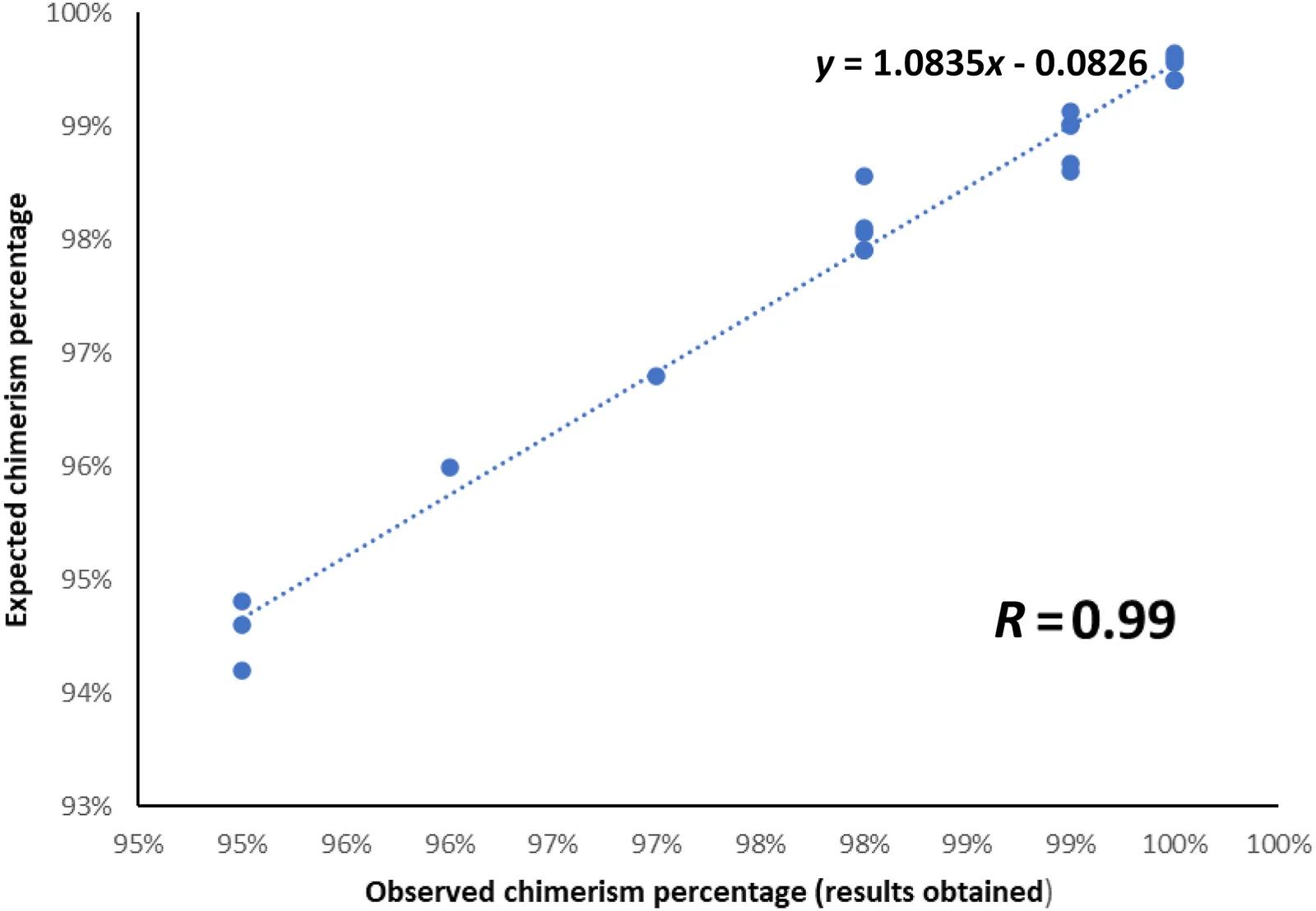
Correlation between the expected and observed chimerism results in mixes from two donor-recipient pairs. Unfractionated cells were used from the first pair, and myeloid cell, T cell, B cell, and natural killer cell fractions were used from the second pair.

Because samples from patients may have some abnormalities in cell counts and other complexities, it was decided to test chimerism from mixes prepared from two actual donor-recipient pairs. For this purpose, five different D/R ratios were prepared from unfractionated cells from the first pair. From the second pair, four mixes with different D/R ratios were prepared from the T cells, B cells, and myeloid fractions, and three mixes with different D/R ratios were prepared from the NK cell fraction. In these mixes, the limit of sensitivity of the assay was tested by including mixes with as low as 0.5% of recipient DNA (Table 2). In this experiment, a strong linear positive correlation between the expected and observed chimerism results was also obtained, with *R*= 0.99 (*P*< 0.0001) (Figure 4).

The absolute differences between the intended and the obtained chimerism percentages were <1% for all 20 mixes. Furthermore, only in two mixes were the differences >0.5%, underlining the consistency and reliability of the results.

#### Test Accuracy for Bone Marrow Biopsy Samples

All the experiments described in the previous section, Linearity and Accuracy, were performed using peripheral blood as a source of cells. Subsequently, the accuracy of the assay was examined using cells isolated from BMB from seven patients. Because cell counts from BMB from patients with malignant conditions tend to be low, it was first tested if it was possible to obtain DNA with concentrations and purity adequate for chimerism analysis from T cells and myeloid cells isolated from this source, following the same protocol used for peripheral blood samples.

Once it was verified that the DNA concentration and purity obtained from BMB samples were acceptable, DNA from the myeloid cells isolated from two BMB samples and from the T cells from two other BMB samples was used to prepare mixes with five different D/R ratios for each pair for a total of 10 mixes. These D/R mixes were used as equivalent to PTx samples. Table 3 shows the comparison between the intended D/R percentages with the actual percentage donor chimerism detected. These results showed a strong positive correlation, with *R* of 0.999 (*P* < 0.00001) for both subsets.

#### Reportable Range

To assess the reportable range of the assay, the seven D/R pairs described in Linearity and Accuracy were used. The ratios used ranged from 99.5% donor and 0.5% recipient through 5% donor and 95% recipient (Tables 1 and 2). A strong linearity was observed with *R* = 0.99 when linear regression analysis was performed (Supplemental Table S2). Therefore, it was concluded that donor-recipient percentage combinations within this range can be accurately determined by this assay.

#### Reproducibility

In a first experiment, the results obtained by two operators from the same laboratory for mixes prepared from healthy individuals were compared. The absolute difference between the results obtained by the two operators ranged between 0.1% and 1.0% (Table 4).

In another experiment, chimerism testing for two mixes prepared from patient and donor DNA samples (D/R ratio of 99%/1%) were repeated 10 times for each pair. The SDs were 0.3% for both samples. The CVs for the minor allele were 39% and 40% for samples 1 and 2, respectively, and 0.3% for the major allele in both samples (Table 5). As expected, the CV significantly increased at lower concentrations because of the inherent loss of precision near the assay's limit of quantification, but it was still considered clinically useful, if the absolute SD was small. Importantly, the repeats were performed by different individuals, also underlining the high reproducibility independently of the operator.

### Comparability Studies

The results obtained in our laboratory were compared with those from two other laboratories. Eight samples were obtained from the HLA laboratory at Loma Linda University Medical Center, and 20 samples were obtained from the Transplant and Genomics Core at Baylor University Medical Center. The results obtained by our laboratory exhibited a strong correlation with those obtained by the other two laboratories, with *R*= 0.99 for both sets of comparisons (Tables 6 and 7).

**Figure 5:**
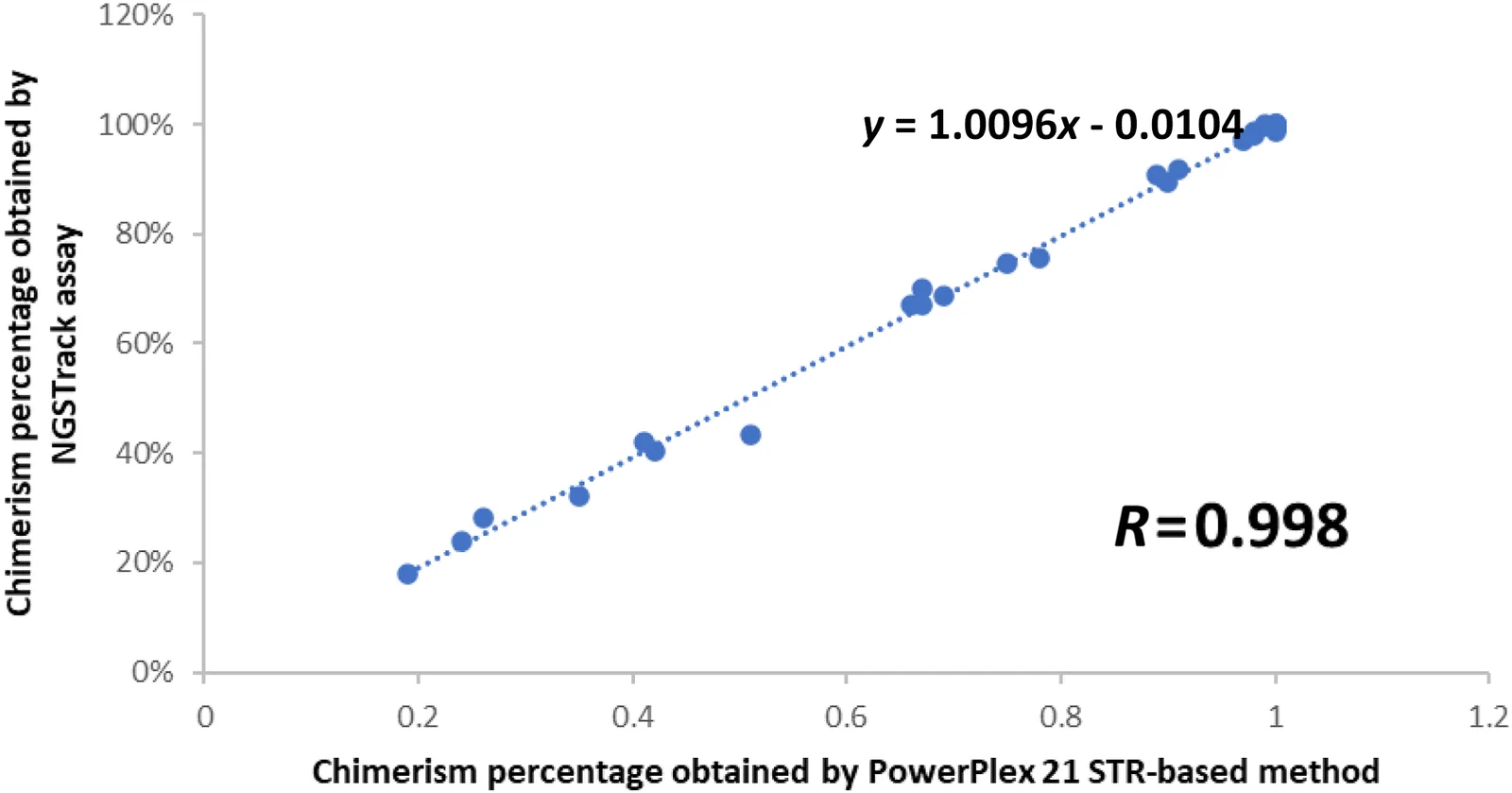
Correlation between chimerism results obtained by NGSTrack assay and PowerPlex 21 short tandem repeat (STR)–based method.

Because STR is the current gold standard method used for chimerism analysis, the study compared our results for 40 samples that were also tested at the Southwest Immunodiagnostics Histocompatibility laboratory using STR-based PowerPlex 21 PCR method. The correlation obtained was 0.99 with no statistical significance difference found between the two methods (*P* = 0.34) when a paired *t*-test was performed (Table 8 and Figure 5).

The absolute difference between the two methods was more noticeable in samples with lower donor chimerism (<95% donor cells) with average difference between the two methods of 1.65% compared with samples with higher donor chimerism (>95% donor cells), where average difference between the two methods was 0.39%. Of the 40 samples, 24 (60%) had >95% donor cells, whereas 16 (40%) had <95% donor cells.

Finally, this study compared our results with the median consensus for five survey samples from American Society of Histocompatibility and Immunogenetics engraftment monitoring proficiency testing program (EMO-1, 2023). A total of 61 laboratories participated in this survey. They used a variety of test platforms. A total of 37 laboratories (60%) used detection of STR using PCR-based methods, and 25 laboratories (40%) used techniques that detect markers other than STR. From them, 20 participants used NGS-based methods, three laboratories used quantitative PCR, one laboratory used digital droplet PCR, and one laboratory used a combination of real-time quantitative PCR when <10% donor was detected and STR PCR-based method when >10% donor was detected. These results highly correlated with the consensus, with *R* = 0.99 (Table 9).

### Informative Markers

This study determined the average number of informative markers used by this assay in a cohort of 30 donor-recipient pairs. From them, 14 donor-recipient pairs were related, and 16 were unrelated. It was found that, on average, 11.6 and 16.5 informative markers were identified and used by the algorithm, respectively, in these groups, ranging between 5 and 15 for the related pairs and between 7 and 27 for the unrelated pairs.

As expected, less informative markers were identified in average for the related pairs than for the unrelated pairs (Supplemental Table S3). The difference in number of informative markers identified between the two groups was statistically significant (*P* < 0.004) (Supplemental Table S3). In all cases, more than three informative markers were identified, which is the current recommendation, according to the UK National External Quality Assessment Service for Leucocyte Immunophenotyping Chimerism Working Group and other expert groups.^1^^,^^2^

Studies have suggested that the distribution of biallelic indels may be associated with race and ethnicity.^19^, ^20^, ^21^, ^22^ From the 30 pairs examined, 19 patients were White, 10 were African American, and 1 was Hispanic. No significant difference was found between the number of informative markers in White patients versus those found in African American patients (*P* = 0.09), suggesting that even if some of the markers may be more frequently found in individuals of a specific race, the assortment of markers used by this assay was well distributed among, at least, these two populations (Supplemental Table S3). However, given the relatively small number of pairs assessed, no definitive conclusions can be made.

### Examples of Clinical Utilization

To illustrate the clinical utilization of this assay, three cases with different patterns of engraftment are presented.

**Figure 6:**
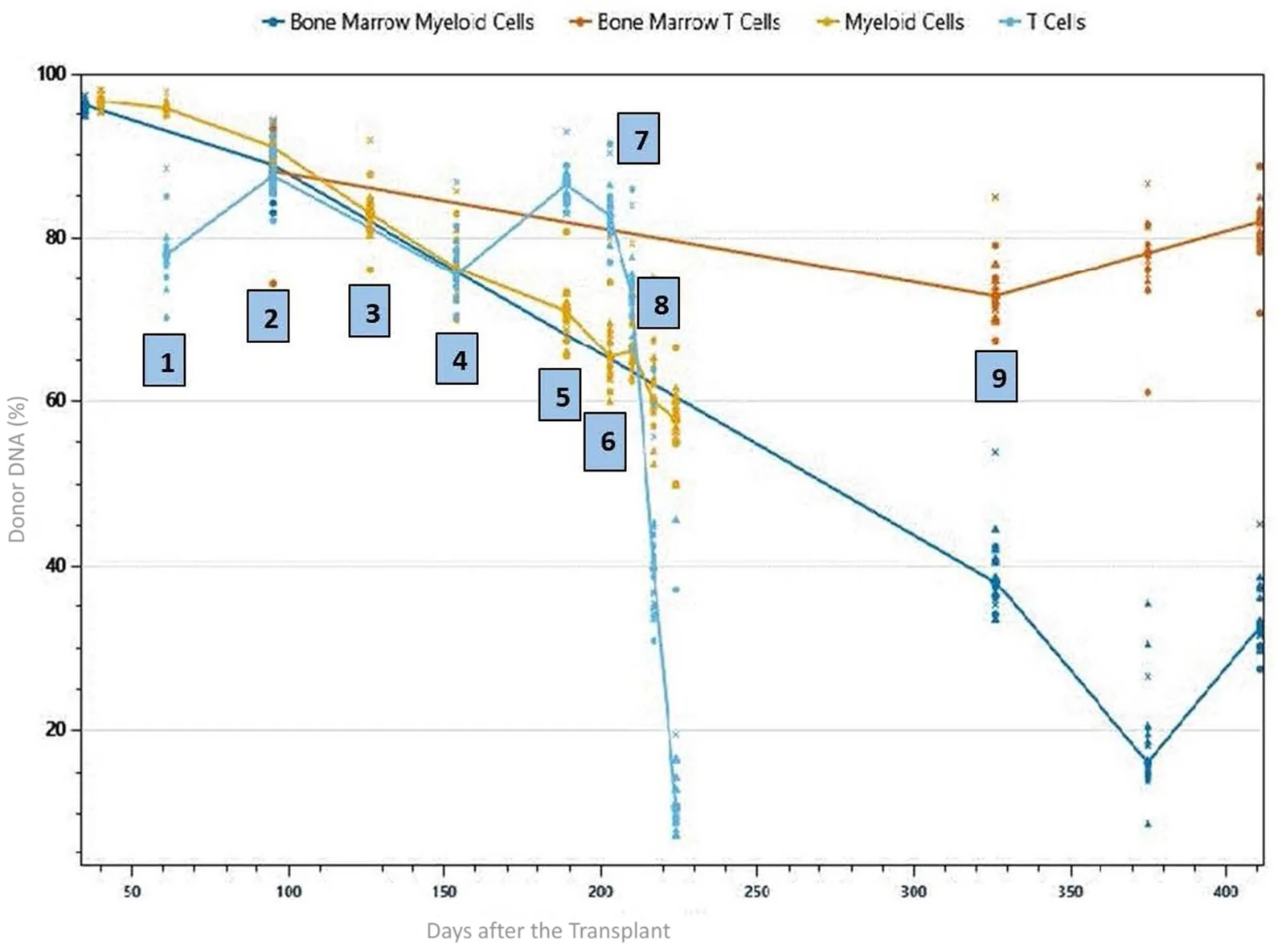
Correlation between the chimerism results with clinical events for Case 1. The light colors correspond to tests performed using peripheral blood samples, and the dark colors correspond to test performed using bone marrow biopsy samples. Each figure (circle, triangle, and cross) in the time points represents a single value for a specific marker. Specifically, markers that are informative for the donor are represented by circles, markers that are informative for the recipient are represented by triangles, and markers that are informative for both are represented by crosses. The **line** that connects different time points is based on the median value of all markers for a specific time point. Numbered points show where tests were performed.

Case 1 is a 26-year–old Hispanic man diagnosed with T-cell acute lymphoblastic leukemia who was transplanted with a 10:10 HLA match unrelated donor. The pair had double permissive *DPB1* mismatches and a minor ABO incompatibility. The cytomegalovirus status was negative for the patient and positive for the donor. Twenty informative markers were identified for this pair. Figure 6 shows different points where the chimerism test was performed. Point 1 corresponds to initial chimerism after the transplant. At day 95 PTx (point 2), chimerism testing showed a decrease in donor chimerism percentage for the myeloid cell fraction, going from 96.6% to 90.9%. At this time, minimal residual disease assessed by flow cytometry using a bone marrow aspirate sample was negative, and no disease progression was detected on a computed tomography scan, but soon after, the patient was diagnosed with fungal pneumonia. Therefore, in the following days, tacrolimus was stopped. Consistent with these developments, chimerism analysis at day 126 PTx (point 3) showed a further decrease in donor chimerism for the myeloid cell fraction, with 82.9%. Chimerism for the T-cell fraction was not possible to assess because of low T-cell counts that yielded insufficient DNA amount. At that time, the patient started treatment with Leukine (sargramostim) three times per week. Chimerism analysis was performed at days 154 (point 4), 189 (point 5), 203 (point 6), 217 (point 7), and 224 (point 8) PTx, observing a persistent decrease in donor cells in both myeloid and T-cell fractions. However, there was a higher decrease in T cells. At day 224, PTx relapse was confirmed by flow cytometry, where 65% of aberrant T cells were detected. The chimerism for T cells in a sample from this day was 10%. The patient then started treatment with pegasparaginase. Chimerism analysis was performed at day 326 (point 9) PTx using a sample from bone marrow biopsy, showing a decrease in donor cells in the T-cell fraction with 72.8% as well as in myeloid fraction with 38.1%. This was consistent with results from a concurrent computed tomography scan that showed mass-like conglomerate lymphadenopathy concerning disease progression.

**Figure 7:**
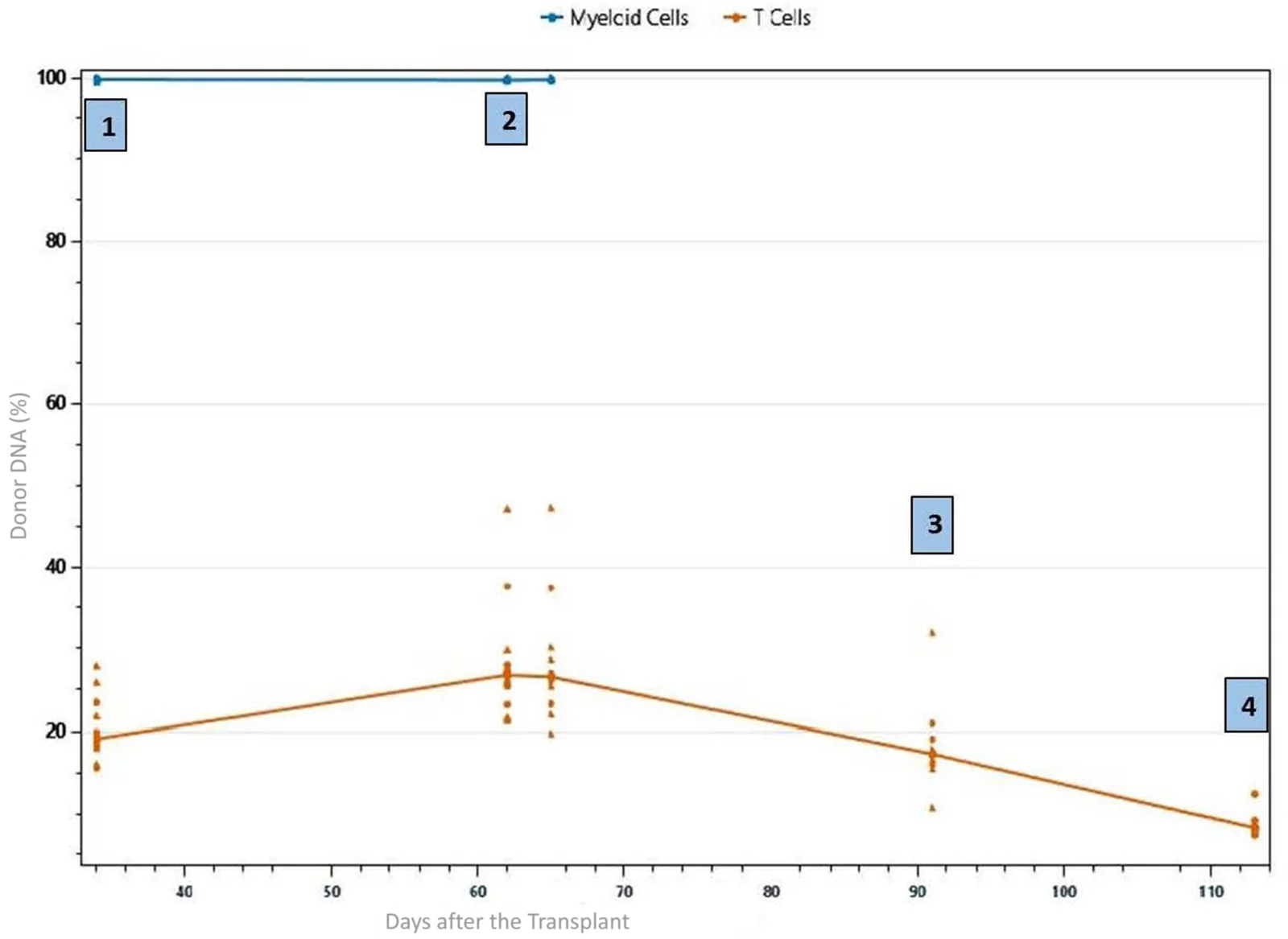
Correlation between the chimerism results with clinical events for Case 2. All the tests were performed using peripheral blood samples. Each figure (circle, triangle, and cross) in the time points represents a single value for a specific marker. Specifically, markers that are informative for the donor are represented by circles, markers that are informative for the recipient are represented by triangles, and markers that are informative for both are represented by crosses. The **line** that connects different time points is based on the median value of all markers for a specific time point. Numbered points show where tests were performed.

Case 2 is a 67-year–old African American man diagnosed with chronic myelomonocytic leukemia that progressed to acute myeloid leukemia. He received a one haplotype matched related transplant from his female child, and the mismatched DP antigen was nonpermissive. The patient did not have any positive HLA antibodies. Both recipient and donor were cytomegalovirus positive, and the ABO for the pair matched. Figure 7 shows the points where the test was performed and the kinetics of chimerism for this patient. A month after the transplant (point 1), chimerism analysis revealed 99.7% and 19% of donor cells for myeloid and T-cell fractions, respectively. Almost simultaneously with these results, he was diagnosed with acute GVHD, further confirmed with duodenal mucosa cytology. A month later (point 2), white blood cell counts were significantly low and never recovered. Chimerisms in the sample concurrent with this event were 99% and 26% of donor cells for myeloid and T-cell fractions, respectively. In a sample from the following month, corresponding to 91 days PTx (point 3), donor cells were 17% for T-cell fraction. Chimerism in the myeloid fraction was impossible to assess because of extremely low cell counts that yield insufficient DNA. Because the patient was a man and his donor a woman, fluorescence *in situ* hybridization analysis of the X and Y chromosomes was performed. In this test, only 26% of female cells were detected, corroborating chimerism results. At that point, immunosuppression had to be stopped because of BK viremia. A month later (point 4), donor cells in the T-cell fraction were only 8% and not possible to assess in the myeloid fraction because of low cell counts, correlating with only 15% of female cells in fluorescence *in situ* hybridization XY test. At that point, the patient became transfusion dependent and sadly expired a month later because of a cardiopulmonary failure caused by acute myeloid leukemia relapse.

**Figure 8:**
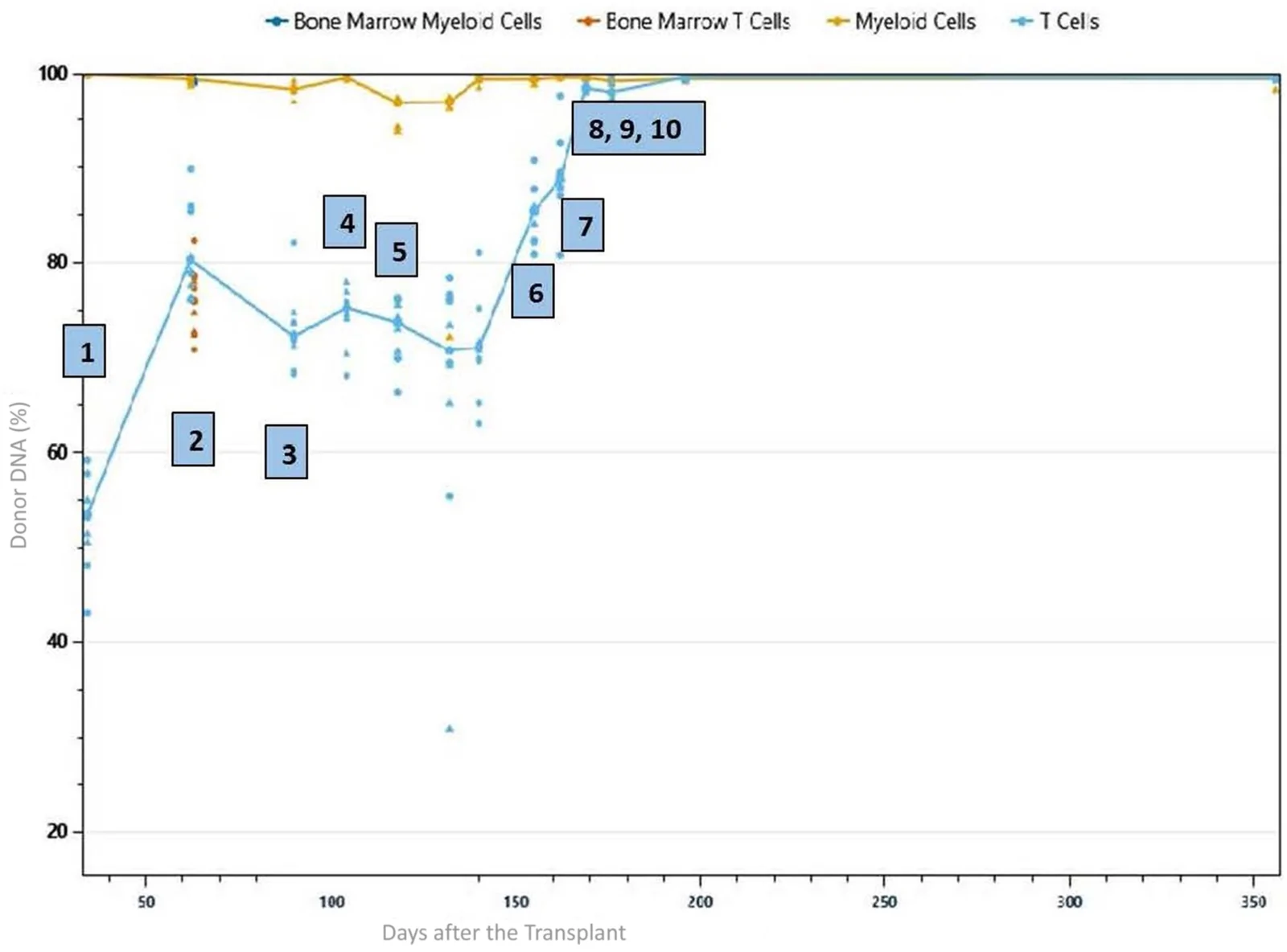
Correlation between the chimerism results with clinical events for Case 3. The light colors correspond to tests performed using peripheral blood samples, and the dark colors correspond to test performed using bone marrow biopsy samples. Each figure (circle, triangle, and cross) in the time points represents a single value for a specific marker. Specifically, markers that are informative for the donor are represented by circles, markers that are informative for the recipient are represented by triangles, and markers that are informative for both are represented by crosses. The **line** that connects different time points is based on the median value of all markers for a specific time point. Numbered points show where tests were performed.

Case 3 is a 40-year–old African American woman diagnosed with acute myeloid leukemia. She received a full 12:12 matched related transplant from a female sibling. This pair had a minor ABO incompatibility because the recipient was AB type, whereas the donor is B type. There was also a mismatch in the cytomegalovirus status because the recipient was negative, whereas the donor was positive. Figure 8 shows the correlation between the chimerism results and the clinical developments described next. Early after the transplant, at 22 days PTx, she was diagnosed with acute GVHD, for which she was treated with steroids and tacrolimus. The first chimerism test was performed 34 days PTx (point 1). It showed 99.9% donor chimerism for myeloid cells and 53.5% for T cells. The next sample at 62 days PTx (point 2) revealed an increase in chimerism for T cells, increasing to 80.3% consistent with improvement in the acute GVHD symptoms. However, in subsequent samples, the percentage chimerism started decreasing for both myeloid and T-cell fractions (points 3 and 4). In a sample from 118 days PTx (point 5), the myeloid cells were 96.8% and the T cells were 73.7% donor. These results were concurrent with the recurrence of GVHD symptoms, such as skin rash and diarrhea, after the initial improvement. The patient was treated for this condition, and after a month, the symptoms started to subside. A sample from 155 days PTx (point 6) showed increased donor chimerism, going back to 99.4% for myeloid cells and 85.4% for T cells. In successive samples (points 7 through 10), donor chimerism percentage continued increasing, correlating with the resolution of the symptoms experienced by the patient.

## Discussion

Chimerism testing is an important diagnostic tool to assess the status of engraftment on patients after they receive allogeneic hematopoietic transplants.^23^^,^^24^ Monitoring chimerism over time provides critical information about the status of immune reconstitution as well as the likelihood of complications, including delayed engraftment, relapse, and graft failure.^25^, ^26^, ^27^, ^28^

In this study, the performance characteristics of the NGStrack assay from GenDx were validated. This assay uses 34 indel biallelic markers to distinguish between recipient and donor. Hence, reads obtained by NGS can have two configurations. Either they are short reads corresponding with the presence of a deletion or long reads when insertion is present.

In contrast, commercial kits that use STR markers typically have between 10 and 21 multiallelic STR markers. Although the difference in number of markers assessed may be considered modest, the higher number of indel markers of the GenDx assay studied here may offer some advantages. First, higher number of markers increases informativity, which is especially important in cases where the recipient and the donor are closely related, and therefore the likelihood of finding sufficient differences among them decreases. This is demonstrated by the comparison between these methods performed in this study, where 13.2 informative markers were found on average in the five pairs compared, when NGSTrack assay was used, whereas only 2.8 informative markers were found on average when PowerPlex 21 was used (Supplemental Table S4).

Another advantage might be enhanced quantitative accuracy because more markers can be used, which helps reduce the effect of stochastic events in the overall result, leading to more precise quantification of chimerism levels. Furthermore, accuracy may also be improved by reducing the impact of false negatives caused by deletions of certain fragments of the genome or even entire chromosomes, which may happen as a result of accumulation of mutations in malignant cells. Because more markers in different regions in the genome are used, these false negatives may be more easily identified and can be excluded from the average result. Improved sensitivity may also result from using more markers because it contributes to accurately detecting low levels of chimerism. This is of crucial importance to diagnose relapse and minimal residual disease.

Finally, the use of biallelic markers may contribute to enhance sensitivity and accuracy when compared with the use of multiallelic markers, such as STR. Increased sensitivity to detect low-level residual recipient cells was consistent in the comparison between methods performed in this study. The chimerism percentages for sample 2 date 1 myeloid, sample 2 date 2 myeloid, and sample 2 date 2 unfractionated were 100% for all, when PowerPlex 21 was used, whereas they were 99.4%, 99.9%, and 98.8%, respectively, when NGSTrack was used (Table 8). Hence, the overall trend across these comparisons supports increased sensitivity of the NGS method.

For all these reasons, the use of indels to monitor chimerism offers enhanced diagnostic capabilities.^29^, ^30^, ^31^, ^32^

Single-nucleotide polymorphisms are variations in only one nucleotide, whereas indels include a longer sequence of nucleotides. The larger size of indels may provide more distinctiveness, or in other words, best discrimination between alleles, which improves quantification accuracy of low-level residual recipient cells. Hence, a smaller number of markers may be necessary to achieve informativity. This may help reduce the complexity of the assay design.^30^^,^^31^^,^^33^

This study found a good consistency of measurements in the reportable range supported by the strong linearity between the expected and observed chimerism results obtained in all the experiments performed. It was determined that the assay can accurately detect and quantify DNA contributed by donor and recipient within the range from 0.5% to 99.5%.

Reproducibility was demonstrated by the strong correlation observed in the results obtained by different operators in the same laboratory and by repetitions performed by different individuals. High precision and accuracy were demonstrated by comparing results with other laboratories using different methods.

Immune reconstitution after hematopoietic transplants is a gradual process, with different cell populations recovering following differential time patterns.^34^ Neutrophils are the first cell population to reconstitute in the early weeks after the transplant, followed by platelets and red blood cells. NK cells are next, between 30 and 100 days PTx, then T cells at around 100 days PTx, and finally B cells, which may take 1 or 2 years to totally reconstitute.^35^, ^36^, ^37^ Given these kinetics, studies have shown that monitoring chimerism in lineage-specific cell subsets provides increased sensitivity over analysis of whole blood chimerism, which translates into early detection of relapse, graft rejection, and other potential complications. It also enhances specificity, allowing for a more accurate interpretation based on the specific clinical situation.^38^, ^39^, ^40^

For this reason, this study assessed the performance of the assay in T cells, B cells, NK cells, and myeloid fractions. It was confirmed that it is possible to obtain DNA with adequate concentration and quality from these cell fractions. However, obtaining DNA with these requirements may be more challenging for B cell and NK cell fractions, consistent with the fact that these cell types are present in lower concentrations in blood compared with other immune cells, such as myeloid and T cells.^41^

Given the strong correlation observed between results obtained with NGSTrack and with the STR-based method, it was concluded that the NGSTrack assay can be used in replacement of more conventional and current gold standard STR-based methods. The reported sensitivity limit for STR-based methods is 5%, whereas the sensitivity limit reported for the indel NGS-based method assessed here is between 0.1% and 1%, depending on the amount of DNA used in the analysis.^1^^,^^4^^,^^9^^,^^31^^,^^42^, ^43^, ^44^ The difference in sensitivity between the two methods was confirmed, which was most noticeable in samples with lower donor chimerism (<95% donor cells).

The optimal number of informative markers sufficient to allow discrimination between recipient and donor has not been well established.^2^^,^^30^^,^^45^ It was decided to direct attention to the average number of markers used by the assay to assess the percentage of cases where informativity was achieved using a cohort of 30 pairs. It was determined that in all cases (100%) there were enough markers identified by the assay to meet and surpass the current consensus and guidelines to monitor at least three informative markers.^1^^,^^45^

Minor allele differences may vary within races and ethnicities.^21^^,^^43^ Hence, the informativity of markers and marker panels may not be the same for pairs depending on the races and ethnicities of recipients and donors. No significant difference was found in the number of informative markers identified for White and African American patients, evidencing that the panel of markers used by this assay is well represented, at least, among these populations.

Of interest, TRKengine software is able to manage analysis of multiple donors. This is a valuable feature for patients who have received multiple transplants from different donors or in cases where donor cells are from cord blood units, where it is frequent to use two units from different donors to reach the adequate number of cells to be transplanted.^46^, ^47^, ^48^

Importantly, although chimerism is a high-complexity testing, this assay follows a relatively simple workflow. It allows for results to be ready the next day after the sample is received with only around 75 minutes of hands-on time. The assay is compatible with Illumina sequencing platforms. Given that several histocompatibility clinical laboratories already use these sequencing platforms to perform HLA typing, it is reasonable to anticipate a rather uncomplicated implementation of this assay in such laboratories. However, based on the relative simplicity of PCR-based techniques, some laboratories may find it difficult to switch to NGS-based techniques, especially in laboratories where they are solely used for chimerism testing.

This assay has some specifications and limitations worth considering. First, it was confirmed that DNA concentrations and quality are key elements to obtain accurate results. Accuracy was compromised in almost all the samples with DNA concentration and quality below the ranges specified by the vendor. The NGStrack kit does not include specific positive or negative controls. However, the presence of all 34 markers in the sequencing run is the best indication of the success of the run. Furthermore, the software has alternative controls. For instance, once the genotype is established, homozygous markers are used to calculate the noise based on the number of reads assigned to the other variant. Hence, if the sample is typed as homozygous for the long variant, any reads for the short variant identified are considered noise. In the PTx samples, all markers for which both donor and recipient have the same uninformative homozygous genotype are used to calculate the noise. The number of reads identified as the other variant is considered noise. The software flags when noise is >1%.

As the resolution of TRKengine algorithm relies on the identification of short and long fragments, and not of individual heterozygous positions, it may be less sensitive to erroneous homozygous versus heterozygous base callings. Finally, as in any other assay based on NGS, low average total read count negatively influences the sensitivity of the assay.^49^^,^^50^ However, low mappability does not necessarily mean that the data quality is compromised because TRKengine software automatically performs quality checks on the acquired reads and ignores reads that contain low Q30 scores in further analysis.

The clinical relevance of tracking chimerism changes over time should be interpreted in the context of factors such as clinical status, the time after the transplant, and the intensity of the conditioning therapy, among others. Three cases were presented to illustrate the clinical utility of the assay. Multiple PTx samples were tested for each patient and correlated with clinical events, demonstrating how the test can be used to monitor the status of engraftment, the risk for PTx complications, and relapse and to inform clinical management.

In conclusion, the NGSTrack chimerism assay provides accurate results that correlate with those obtained by RT-PCR methods using STR markers and surpass them in sensitivity. It was demonstrated that it is possible to obtain DNA with concentration and purity adequate for chimerism testing using NGSTrack assay from isolated cell subsets and from whole peripheral blood samples, as well as from bone marrow biopsy samples. The relatively simple workflow and short turnaround time of this assay are important advantages in support of its use for monitoring the PTx progression and to inform early clinical interventions. The low DNA input needed for the assay is another advantage, especially in cases of patients with hematological conditions that cause low cell counts. It was corroborated that the number of markers used by the assay is adequate to obtain informativity according to current recommendations and is well represented among some of the major racial groups. Finally, through the discussion of three clinical cases, the precise correlation between the chimerism results with clinical events was shown, which validates the usefulness of the testing for early diagnosis, clinical management, and interventions.

## Disclosure Statement

GenDx, which is the company that developed and distributes the assay evaluated in this study, had agreed to help the authors pay the publication fees required by this journal. We confirm that GenDx had no involvement in the analyses, results, or points of discussion considered in our study.
