# Supplementary material for: Evaluation of Chimerism Testing by Next-Generation Sequencing Using Insertion/Deletion Markers: Analytical Validation and Examples of Clinical Utilization
Source: J Mol Diagn. 2026 Apr 22;28(7):535–52. doi: 10.1016/j.jmoldx.2026.04.002 (PMC13494106; doi:10.1016/j.jmoldx.2026.04.002)
Supplement: Supplementary file 1 [file mmc1.docx]

Supplemental Table S1. DNA concentrations and 260/280 ratios for recipients and donors’ samples for pairs 1 through 5. In bold are DNA concentrations and 260/280 ratios below recommended.

| Pair Recipient - Donor | Sample | DNA Concentration (ng/μL) | 260/280 ratio |
| --- | --- | --- | --- |
| Pair 1 | Recipient Genotyping | 149.3 | 1.82 |
|  | Donor Genotyping | 141.8 | 1.81 |
|  | Recipient T cell (CD3+) | 18.5 | 1.8 |
|  | Donor T cell (CD3+) | 17.8 | 1.89 |
|  | Recipient B cell (CD19+) | 10 | **1.51** |
|  | Donor B cell (CD19+) | **3.1** | **1.58** |
|  | Recipient Myeloid | 32.6 | 1.9 |
|  | Donor Myeloid | 40.8 | **1.61** |
|  | Recipient NK cells (CD56) | 10.9 | 1.93 |
|  | Donor NK cells (CD56 | 12.4 | 2.01 |
| Pair 2 | Recipient Genotyping | 94.6 | 1.8 |
|  | Donor Genotyping | 105.5 | 1.85 |
|  | Recipient T cell (CD3+) | 20.5 | 1.97 |
|  | Donor T cell (CD3+) | 34.3 | **1.79** |
|  | Recipient B cell (CD19+) | **7.3** | 1.89 |
|  | Donor B cell (CD19+) | **2.5** | **1.45** |
|  | Recipient Myeloid | 38 | **1.79** |
|  | Donor Myeloid | 20.8 | 1.92 |
|  | Recipient NK cells (CD56) | **6.7** | **1.39** |
|  | Donor NK cells (CD56 | **4.2** | **1.62** |
| Pair 3 | Recipient Genotyping | 141.8 | 1.81 |
|  | Donor Genotyping | 82.2 | 1.84 |
|  | Recipient T cell (CD3+) | 17.8 | 1.89 |
|  | Donor T cell (CD3+) | **9.3** | **1.76** |
|  | Recipient B cell (CD19+) | **3.1** | **1.58** |
|  | Donor B cell (CD19+) | **2.6** | 1.82 |
|  | Recipient Myeloid | 40.8 | **1.61** |
|  | Donor Myeloid | 30.9 | 1.82 |
|  | Recipient NK cells (CD56) | 12.4 | **1.01** |
|  | Donor NK cells (CD56 | 10.9 | **1.39** |
| Pair 4 | Recipient Genotyping | 105.5 | 1.85 |
|  | Donor Genotyping | 94.6 | 1.8 |
|  | Recipient T cell (CD3+) | 34.3 | **1.79** |
|  | Donor T cell (CD3+) | 20.5 | 1.97 |
|  | Recipient B cell (CD19+) | **2.5** | **1.45** |
|  | Donor B cell (CD19+) | **7.3** | 1.89 |
|  | Recipient Myeloid | 20.8 | 1.92 |
|  | Donor Myeloid | 38 | **1.79** |
|  | Recipient NK cells (CD56) | **4.2** | **1.62** |
|  | Donor NK cells (CD56 | 12.4 | 2.01 |
| Pair 5 | Recipient Genotyping | 82.2 | 1.84 |
|  | Donor Genotyping | 149.3 | 1.82 |
|  | Recipient T cell (CD3+) | **9.3** | **1.76** |
|  | Donor T cell (CD3+) | 18.5 | 1.8 |
|  | Recipient B cell (CD19+) | **2.6** | 1.82 |
|  | Donor B cell (CD19+) | 10 | 1.51 |
|  | Recipient Myeloid | 30.9 | 1.82 |
|  | Donor Myeloid | 32.6 | 1.9 |
|  | Recipient NK cells (CD56) | 10.9 | **1.39** |
|  | Donor NK cells (CD56 | 10.9 | **1.39** |
