# Supplementary material for: Evaluation of Chimerism Testing by Next-Generation Sequencing Using Insertion/Deletion Markers: Analytical Validation and Examples of Clinical Utilization
Source: J Mol Diagn. 2026 Apr 22;28(7):535–52. doi: 10.1016/j.jmoldx.2026.04.002 (PMC13494106; doi:10.1016/j.jmoldx.2026.04.002)
Supplement: Supplementary file 2 [file mmc2.docx]

Supplemental Table S2. Reportable range of the assay based on testing of 7 D/R pairs with different D/R ratios ranging from 99.5% donor and 0.5% recipient through 5% donor and 95% recipient.

| **Mixed Samples** | **Cell type** | **Intended Percentage** | **Chimerism Result obtained** |
| --- | --- | --- | --- |
| Recipient 1 - Subject 1 | Unfractionated | 5.0% | 4.9% |
| Donor 1- Subject 2 | Myeloid | 5.0% | 5.1% |
|  | T cells (CD3) | 5.0% | 4.8% |
|  | NK cells (CD56) | 5.0% | 5.8% |
|  | B cells (CD19) | 5.0% | 14.3% |
| Recipient 2 - Subject 3 | Unfractionated | 25.0% | 25.7% |
| Donor 2 - Subject 4 | Myeloid | 25.0% | 26.2% |
|  | T cells (CD3) | 25.0% | 32.9% |
|  | NK cells (CD56) | 25.0% | 32.5% |
|  | B cells (CD19) | 30.0% | 30.8% |
| Recipient 3 - Subject 2 | Unfractionated | 50.0% | 50.5% |
| Donor 3 - Subject 5 | Myeloid | 50.0% | 48.9% |
|  | T cells (CD3) | 50.0% | 49.7% |
|  | NK cells (CD56) | 50.0% | 47.6% |
|  | B cells (CD19) | 50.0% | 56.5% |
| Recipient 4 - Subject 4 | Unfractionated | 75.0% | 74.9% |
| Donor 4 - Subject 3 | Myeloid | 75.0% | 74.8% |
|  | T cells (CD3) | 75.0% | 75.5% |
|  | NK cells (CD56) | 75.0% | 75.4% |
|  | B cells (CD19) | 75.0% | 76.2% |
| Recipient 5 - Subject 5 | Unfractionated | 95.0% | 94.8% |
| Donor 5 - Subject 1 | Myeloid | 95.0% | 94.2% |
|  | T cells (CD3) | 95.0% | 94.6% |
|  | NK cells (CD56) | 95.5% | 95.7% |
|  | B cells (CD19) | 95.5% | 95.0% |
| Recipient 6 -Patient 1 Donor 6 - Patient 2 | Unfractionated | 95.5% | 94.7% |
|  |  | 95.5% | 96.7% |
|  |  | 95.5% | 95.8% |
|  |  | 96% | 95.98% |
|  |  | 97% | 96.79% |
| Recipient 3 -Subject 2 Donor 7-Subject 6 | T cell (CD3) | 98% | 98.05% |
|  |  | 98.0% | 98.1% |
|  |  | 98.0% | 97.9% |
|  |  | 98.0% | 97.9% |
|  | B cells (CD19) | 98.0% | 98.6% |
|  |  | 99% | 98.66% |
|  |  | 99.0% | 99.0% |
|  |  | 99.0% | 98.6% |
|  | Myeloid | 99.0% | 99.0% |
|  |  | 99.0% | 99.1% |
|  |  | 99.50% | 99.55% |
|  |  | 99.5% | 99.6% |
|  | NK cells (CD56) | 99.5% | 99.4% |
|  |  | 99.5% | 99.4% |
|  |  | 99.5% | 99.6% |
