# Supplementary material for: Evaluation of Chimerism Testing by Next-Generation Sequencing Using Insertion/Deletion Markers: Analytical Validation and Examples of Clinical Utilization
Source: J Mol Diagn. 2026 Apr 22;28(7):535–52. doi: 10.1016/j.jmoldx.2026.04.002 (PMC13494106; doi:10.1016/j.jmoldx.2026.04.002)
Supplement: Supplementary file 3 [file mmc3.docx]

Supplemental Table S3.

Difference between the number of informative markers found in related versus unrelated pairs.

| **Related pairs** | | | **Unrelated pairs** | | |
| --- | --- | --- | --- | --- | --- |
| **Pairs** | **Recipient Race** | **# of Informative markers** | **Pairs** | **Recipient Race** | **# of Informative markers** |
| 1RP | African American | 10 | 1UP | Caucasian | 16 |
| 2RP | Caucasian | 11 | 2UP | Caucasian | 13 |
| 3RP | African American | 12 | 3UP | African American | 16 |
| 4RP | African American | 11 | 4UP | Caucasian | 19 |
| 5RP | Caucasian | 13 | 5UP | Caucasian | 27 |
| 6RP | Caucasian | 5 | 6UP | Caucasian | 21 |
| 7RP | African American | 15 | 7UP | Caucasian | 7 |
| 8RP | African American | 12 | 8UP | Caucasian | 21 |
| 9RP | African American | 10 | 9UP | Caucasian | 9 |
| 10RP | African American | 7 | 10UP | Hispanic | 20 |
| 11RP | Caucasian | 12 | 11UP | Caucasian | 15 |
| 12RP | Caucasian | 15 | 12UP | Caucasian | 17 |
| 13RP | African American | 15 | 13UP | Caucasian | 20 |
| 14RP | African American | 14 | 14UP | Caucasian | 21 |
|  |  |  | 15UP | Caucasian | 14 |
|  |  |  | 16UP | Caucasian | 8 |
|  |  |  |  |  |  |
|  | **Average # of informative markers** | 11.6 |  | **Average # of informative markers** | 16.5 |

Paired T-test results of comparisons between the number of informative markers found in related versus unrelated pairs and between the number of informative markers found in Caucasians versus African American patients.

| **# of informative markers** | **paired T-test** |
| --- | --- |
| related pairs vs. unrelated pairs | 0.004 |
| Caucasians vs. African Americans | 0.09 |
