# Supplementary material for: Evaluation of Chimerism Testing by Next-Generation Sequencing Using Insertion/Deletion Markers: Analytical Validation and Examples of Clinical Utilization
Source: J Mol Diagn. 2026 Apr 22;28(7):535–52. doi: 10.1016/j.jmoldx.2026.04.002 (PMC13494106; doi:10.1016/j.jmoldx.2026.04.002)
Supplement: Supplementary file 4 [file mmc4.docx]

Supplemental Table S4. Difference between the number of informative markers found in the five pairs used to compare NGSTrack indel-based assay and PowerPlex 21 STR-based assay.

| **Pair** | **Number of informative markers** | |
| --- | --- | --- |
|  | **PowerPlex21 assay** | **NSGTrack assay** |
| Pair 1 | 2 | 8 |
| Pair 2 | 5 | 17 |
| Pair 3 | 3 | 6 |
| Pair 4 | 1 | 19 |
| Pair 5 | 3 | 16 |
| Average | 2.8 | 13.2 |
